# The Relationship between Pulmonary Damage and Peripheral Vascular Manifestations in Systemic Sclerosis Patients

**DOI:** 10.3390/ph14050403

**Published:** 2021-04-23

**Authors:** Barbara Ruaro, Marco Confalonieri, Francesco Salton, Barbara Wade, Elisa Baratella, Pietro Geri, Paola Confalonieri, Metka Kodric, Marco Biolo, Cosimo Bruni

**Affiliations:** 1 Department of Pulmonology, University Hospital of Cattinara, 34149 Trieste, Italy; marco.confalonieri@asugi.sanita.fvg.it (M.C.); francesco.salton@gmail.com (F.S.); pietrogeri@gmail.com (P.G.); paola.confalonieri.24@gmail.com (P.C.); metka.kodric@asuits.sanita.fvg.it (M.K.); marcobiolo@gmail.com (M.B.); 2 AOU City of Health and Science of Turin, Department of Science of Public Health and Pediatrics, University of Torino, 10126 Torino, Italy; barbarawade@hotmail.com; 3 Department of Radiology, Cattinara Hospital, University of Trieste, 34149 Trieste, Italy; elisa.baratella@gmail.com; 4 Department of Experimental and Clinical Medicine, Division of Rheumatology, University of Firenze, 50121 Florence, Italy; cosimobruni85@gmail.com

**Keywords:** systemic sclerosis, pulmonary involvement, microvascular involvement, pulmonary arterial hypertension, interstitial lung disease, nailfold capillaroscopy

## Abstract

Systemic sclerosis (SSc) is an autoimmune disease, characterized by the presence of generalized vasculopathy and tissue fibrosis. Collagen vascular disorder in SSc is due to fibroblast and endothelial cell dysfunctions. This leads to collagen overproduction, vascular impairment and immune system abnormalities and, in the last stage, multi-organ damage. Thus, to avoid organ damage, which has a poor prognosis, all patients should be carefully evaluated and followed. This is particularly important in the initial disease phase, so as to facilitate early identification of any organ involvement and to allow for appropriate therapy. Pulmonary disease in SSc mainly involves interstitial lung disease (ILD) and pulmonary arterial hypertension (PAH). High-resolution computed tomography (HRCT) and pulmonary function tests (PFT) have been proposed to monitor parenchymal damage. Although transthoracic echocardiography is the most commonly used screening tool for PAH in SSc patients, definitive diagnosis necessitates confirmation by right heart catheterization (RHC). Moreover, some studies have demonstrated that nailfold videocapillaroscopy (NVC) provides an accurate evaluation of the microvascular damage in SSc and is able to predict internal organ involvement, such as lung impairment. This review provides an overview of the correlation between lung damage and microvascular involvement in SSc patients.

## 1. Introduction

Systemic sclerosis (SSc), a heterogeneous disease, is characterized by immune dysfunction, often leading to organ damage due to inflammation, endothelial dysfunction and fibrosis [1,2,3,4]. SSc involves microcirculation structural and functional alterations [5,6,7,8,9]. The main cause of death in SSc patients is not only collagen overproduction but also the effects collagen overproduction has on the pulmonary system. This includes fibrosis or pulmonary artery hypertension (PAH) [10,11,12,13,14,15]. Recent guidelines have recommend screening with high resolution computed tomography (HRCT) to diagnose interstitial lung diseases (ILD) in SSc patients at the baseline visit and once the diagnosis of ILD has been established [16,17], whilst a combination of HRCT and pulmonary function tests is recommended to quantify the extent and severity of ILD [16,17,18,19]. Screening for PAH in SSc is transthoracic echocardiography, which has a sensitivity of 90%, even if definitive diagnosis is to be confirmed by right heart catheterization (RHC) [20,21,22,23,24,25]. Although nailfold videocapillaroscopy (NVC) is the validated method for assessing peripheral vascular damage [26,27,28,29], several studies have demonstrated that NVC is capable of predicting internal organ involvement [27,28,29,30,31,32,33,34,35].

This review aims at providing updated information on the link between pulmonary damage, i.e., ILD and PAH, and peripheral vascular manifestations, evaluated by NVC, in SSc patients.

## 2. Pulmonary Manifestations

Pulmonary disease in SSc includes interstitial lung disease (ILD) and pulmonary arterial hypertension (PAH) [36,37,38]. All SSc patients should be screened to detect any ILD and PAH development, at diagnosis and periodically thereafter. Indeed, although there has been no statistically significant change in the SSc mortality rate over the past 40 years, the proportion of deaths due to ILD and PAH has increased [36,37,38,39,40,41]. ILD and PAH are the two main causes of death in SSc patients and account for 33% and 28% of deaths, respectively [36,37,38,39,40,41]. Although ILD is reported to be more common in diffuse cutaneous SSc (dcSSc) whilst PAH is reportedly more common in limited cutaneous SSc (lcSSc), both pulmonary manifestations have been described in each of the disease subsets. Patients with rarer phenotypes associated with antiTh/To and anti U3RNP antibodies may have PAH and ILD concomitantly. Pulmonary disease may even occur in SSc with no skin involvement, i.e., scleroderma sine scleroderma [36,37,38,39,40,41]. 

Although the clinical course varies from mild and asymptomatic to severely debilitating, most patients have some degree of pulmonary fibrosis [39,40]. The most common early symptoms related to SSc pulmonary manifestations are exertional dyspnea and dry cough and, in most cases, are non-specific findings [42,43,44,45,46]. Should this be the case, then a differential diagnosis should be made to investigate/exclude SSc-ILD, PAH, deconditioning, chronic anemia and/or left heart involvement with a reduced or preserved ejection fraction [42,43,44,45,46]. Considering the frequency of lung involvement in SSc and its impact on prognosis, early recognition of lung involvement and prompt appropriate treatment is a must [36,37,38,39,40,41]. Although there are inherent challenges in the management of both PAH and ILD, with the early diagnosis, treatment may have a higher chance of efficacy for each of these lung complications [2,10].

The etiologic or enhancing factors of pulmonary involvement in SSc patients are still a question of debate. A previous study implicated genetic factors, i.e., HLA class II (1–3). Others have implicated immunologic factors for which certain autoantibodies such as anti–topoisomerase I (anti–topo I) may be markers [1,2,3]. The few studies that have addressed the impact of race or ethnicity on lung involvement in early SSc suggest a worse prognosis for nonwhite groups (e.g., African Americans, the Japanese population and Choctaw Indians). However, ethnicity is not only defined by racial or genetic factors but also by sociodemographic and cultural factors [1,2,3,4].

## 3. Interstitial Lung Disease 

ILD complicates diffuse cutaneous SSc (dcSSc) in 53% of cases but may also be associated in 35% of cases with limited cutaneous SSc (lcSSc), as reported by the European Scleroderma Trials and Research group (EUSTAR) [39,42]. Furthermore, several autopsy studies reported that parenchymal involvement, in the form of ILD, was present in up to 90% of SSc patients. Risk factors for ILD development include African American ethnicity, skin score, serum creatinine and creatine phosphokinase levels, hypothyroidism and cardiac involvement [1,2,42,43,44]. Genetic factors, specific serological findings and anti-topoisomerase and anti-endothelial cell antibodies can predict the presence of lung involvement [1,2,42,43,44]. It is also reported that the patients with dcSSc have a higher incidence of interstitial disease [1,2,3]. Predictors of severe restrictive lung disease (defined by a forced vital capacity (FVC) of 50% predicted) include African American ethnicity, male gender, the degree of physiological abnormalities at diagnosis (FVC and diffusing lung capacity for carbon monoxide (DLCO)) and a younger age [1,2,46,47,48].

Unfortunately, there are limited treatment options for this manifestation. This is due to the paucity of high-quality, randomized, controlled trials that specifically target SSc-ILD. Moreover, historically, studies have favored cyclophosphamide (CYC) for SSc-ILD treatment, as also suggested in the most recent European League against Rheumatism (EULAR) recommendations [2,49]. Supportive data have shown that nintedanib, a multi-tyrosine kinase inhibitor, and tocilizumab (TCZ) significantly inhibit the progressive functional decline [2,49]. Current innovative proposals have also recently been made on the basis of clinical and preclinical evidence for rituximab (RTX) and pirfenidone (PIRF), as well as hematopoietic stem cell and lung transplantation [2,49]. However, the safety and efficacy of emerging experimental therapies for SSc-ILD do require further investigation.

Other findings were that high-resolution computed tomography (HRCT) evidenced interstitial abnormalities in as many as 90% of patients, and 40–75% had changes in pulmonary function tests (PFT) [46,47,48,49,50,51,52]. 

## 4. Imaging 

SSc-ILD is diagnosed by HRCT, which is a simple non-invasive, sensitive investigation, able to detect parenchymal lung disease [53,54,55,56,57,58,59,60]. However, despite its high sensitivity, HRCT may be normal in some patients with pulmonary function test abnormalities or abnormal chest auscultation (i.e., crackles) [46,47,48,58]. The absence of lung involvement in HRCT at the time of disease presentation may lower the long-term risk of developing SSc-ILD, as 85% of patients have a normal HRCT at an average 5 year follow-up [46,47,48,49,50,51,52,53,54,55]. These factors stress the importance of making an SSc-ILD diagnosis by combining clinical findings, pulmonary function tests and HRCT abnormalities. 

**Figure 1:**
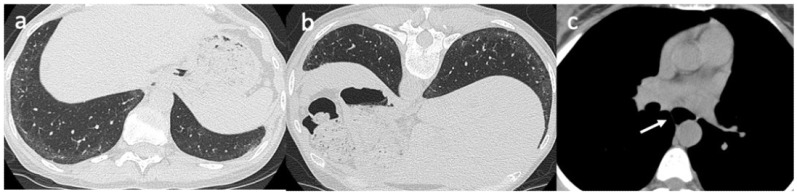
A 43-year-old female with a diagnosis of systemic sclerosis. Axial high-resolution CT scan obtained in the supine position shows subtle ground-glass opacities in the subpleural regions of the lung bases, suspicious for NSIP (non-specific interstitial pneumonia) (**a**). When these findings are not prominent, an additional scan can be acquired in the prone position to differentiate ground-glass opacities due to gravitational phenomena from interstitial lung disease. In this patient the ground-glass opacities persist in the prone position, confirming the interstitial lung involvement (**b**). Additional systemic sclerosis-related findings should be searched for, such as a dilated esophagus on images reconstructed using an appropriate mediastinal window setting (**c**).

A common HRCT pattern of SSc-ILD is characterized by a greater proportion of ground-glass opacities with a lower degree of reticulation, suggestive of nonspecific interstitial pneumonia (NSIP). The predominant observations in the basal areas of the lungs are low lung volumes and interstitial reticular thickening. In the late lung involvement stages, pulmonary fibrosis manifests as traction bronchiectasis and honeycomb cysts, a marker for usual interstitial pneumonia (UIP) [55,56,57,58,59,60,61,62,63,64] (Figure 1). These two alterations have been observed in up to 33% of SSc-ILD patients, suggesting that these patients may have a mixture (or overlap) of UIP and NSIP patterns [55,56,57,58,59,60,61,62,63,64]. 

Even when treated, ground-glass opacities progress to fibrosis and lead to honeycombing/traction bronchiectasis and/or bronchiectasis formation over time in up to 60% of patients [55,56,57,58,59,60,61,62,63,64]. There is a correlation between ground-glass opacities/consolidation and active inflammation, whilst reticular opacities/honeycombing correlate with fibrotic lesions. There is a better treatment response in patients with HRCT features of ground-glass opacities, as they are markers of inflammation and reversible lung injury [50,51,52,64,65,66,67]. 

Although the HRCT pattern correlates well with histology, nowadays lung biopsies are rarely performed, except for the exclusion of other parenchymal processes [52,53,54,55]. However, when performed, histology analysis shows interstitial fibrosis with temporal homogeneity and a modest inflammatory cell infiltrate (i.e., fibrotic NSIP) [42,43,44,52,53,54,55].

It has been observed that HRCT is more sensitive than chest radiography (CR) in diagnosing and characterizing SSc-related lung diseases, as there may be a normal CR in early lung involvement and even in some patients with pulmonary symptoms [58,64,67]. Moreover, ILD HRCT findings correlate more closely with pulmonary function test abnormalities, demonstrating that SSc-related lung injury is a restrictive disorder, associated with low lung volumes, and a diffusion disorder, which impairs carbon monoxide diffusion capacity [55,58]. 

Chest HRCT findings also have prognostic implications in SSc and SSc-ILD. The absence of lung involvement in HRCT at the time of disease presentation is a good long-term prognostic indicator of SSc-ILD [42,64]. Conversely, the presence of SSc-ILD and its extent, quantified by both visual semi-quantitative and software-based quantitative methods, are able to predict disease-related mortality [64,65]. Along with parenchymal features, lung vessels have also been recently investigated on HRCT. It was observed that the extent of lung volume occupied by vessels has a statistically significant correlation with the extent of SSc-ILD, ILD-related restrictive functional changes and decline in the diffusion capacity of carbon monoxide (DLCO) among SSc patients with or without ILD [51,65,66,67].

Recently, various radiation-free modalities have been tested, and it seems that lung MRI may be a promising tool for SSc-ILD detection and prognostication [68]. This may be due to the fact that MRI is capable of differentiating inflammation-predominant versus fibrosis-predominant lesions, offering information as to the choice for more anti-inflammatory or more anti-fibrotic targeting medications [68]. Moreover, ultrasound lung investigations are becoming widespread in the SSc-ILD field due to their potential for ILD screening [69,70,71]. Ultrasound correlates well with ILD extent and lung impairment and has a significant prognostic value in the evaluation of lung involvement, even if further studies are required to support the use of the technique [69,70,71].

## 5. Pulmonary Function Tests

Pulmonary function tests (PFT) are essential, readily available non-invasive tests able to detect SSc-related pulmonary changes. PFT in SSc-ILD is characterized by a restrictive ventilatory defect with a decrease in functional vital capacity (FVC) and/or total lung capacity (TLC), a preserved forced expiratory volume in 1 s (FEV1), a normal or increased FEV1/FVC ratio and a DLCO reduction [72,73,74,75,76,77]. SSc survival has been inversely correlated with the degree of restrictive ventilatory defect on pulmonary function tests. Several studies have reported an 87% 10-year survival rate in patients with minimal to absent restriction and a 75% and 58% 10-year survival rate in patients with moderate or severe restriction [72,73,74,75,76,77]. Both FVC and DLCO have been identified as adverse prognostic markers in SSc-related lung injury. Indeed, almost all patients have a reduced DLCO, along with other pulmonary function test abnormalities. However, a reduced DLCO is the single most significant marker of poor outcome and correlates with the extent of lung disease on HRCT [72,73,74,75,76,77]. An important factor, not to be overlooked, is the fact that although patients with early SSc-ILD may have signs of lung disease at HRCT and a DLCO decrease, they may also have preserved lung volumes [72,73,74,75,76,77].

Recent studies have demonstrated that more than 60% of SSc-ILD patients had normal PFT at HRCT [72,73,74,75,76,77]. Therefore, although PFT is an important diagnostic tool for SSc-ILD, it is not sensitive enough make an early detection [72,73,74,75,76,77]. Regular annual PFT after SSc diagnosis may be useful to evidence any changes in lung function that are indicative of ILD [72,73,74,75,76,77].

The reduced DLCO levels observed in SSc-ILD are due to a variable combination of a reduction in alveolar volume and/or thickening of the alveolar–capillary membrane. Impaired DLCO in SSc-induced lung injury is usually secondary to two main pathological conditions, i.e., ILD and PAH, even if it may be observed without these complications. Indeed, an isolated DLCO impairment, with reduced FVC/TLC and clinical and/or radiological signs of parenchymal lung involvement, has been attributed to lung vasculopathy and could be considered a good prognostic sign, even it may rarely be associated with the future development of PAH or SSc-ILD [72,73,74,75,76,77,78]. 

Up until 2010, the most common outcome test used in clinical lung disease studies was the DLCO evaluation, which was later surpassed by FVC. Indeed, the FVC percentage predicted the primary endpoint in 70.4% of studies, whilst only 11.3% of DLCO evaluations were predictive. To the best of our knowledge, only five studies specifically aimed to validate PFT: two concluded that the extent of SSc-ILD was best measured by DLCO whilst the other three did not favor any PFT parameter. These studies also showed validity measures for total lung capacity (TLC). Despite the current preference for FVC, available evidence suggests that DLCO and TLC should not yet be discounted as potential surrogate markers for SSc-ILD progression [55,72,73,74,75,76,77,78].

## 6. Pulmonary Arterial Hypertension 

The highest prevalence of PAH amongst the various connective tissue diseases is observed in SSc patients, and it may occur in all forms [20,21,22,23,24,25]. The main pathophysiological alteration in SSc-PAH is small vessel vasculopathy [20,21,22,23,24,25,43,51]. This is usually diagnosed 10 to 15 years after SSc onset and is associated with early mortality [20,21,22,23,24,25,51]. PAH was previously defined as an average pulmonary artery pressure (mPAP) of ≥ 25 mmHg, assessed by right heart catheterization (RHC), with an mPAP between 21 mmHg and 24 mmHg, which was considered “borderline pulmonary hypertension” (Bo-PAH) [19,78]. At the 6th World Symposium of Pulmonary Hypertension, PAH was finally defined as an mPAP of ≥ 21 mmHg with a peripheral vascular resistance (PRV) of ≥ 3 Woods Units (WU) [19,78].

The presence of PAH in SSc may be the result of vaso-occlusive pulmonary artery hypertension (SSc-PAH), left ventricular heart dysfunction or pulmonary hypoxic disease, classified as group 1, 2 and 3 PAH, respectively [19,78]. Group 1 includes patients with isolated PAH without ILD, whilst PAH patients with ILD are classified into group 3, in the PH classification [19,78]. 

## 7. Screening 

Our understating of this condition has been changed by the development of systematic algorithms for early diagnosis over the last decade and the data from the follow-up cohorts of incidental SSc-PAH [24]. Indeed, echocardiograph assessment is the most frequently used screening tool to identify candidates for RHC, and a tricuspid regurgitation (TR) velocity of ≥ 2.5 m/s is considered to be highly suggestive of PAH [78]. However, the sensitivity at this TR velocity threshold is limited and misses 20% of mild PAH patients [19,78].

Other studies documented that 55–86% of patients with an echocardiography finding suggestive of pulmonary hypertension (e.g., a right ventricular systolic pressure (RVSP) of 30 to 40 mmHg or higher, with or without symptoms) will have pulmonary hypertension on RHC. When the measurement of RVSP is combined with an increase in right atrial or right ventricular size, reduced pulmonary artery acceleration and decreased right ventricular function, the specificity of echocardiography for pulmonary hypertension diagnosis will be higher [78,79,80,81,82,83,84].

The multi-dimensional DETECT algorithm, the forced vital capacity (FVC)/diffusion capacity for carbon monoxide (DLCO) ratio or N-terminal-pro-brain natriuretic peptide (NT-pro-BNP) are all proven screening tools that support the early diagnosis of SSc-PAH [24,75,79]. Reduced DLCO levels in PAH are due to vascular remodeling, which leads to vessel wall tightening and arterial stiffness. The presence of a baseline isolated marked reduction in DLCO (<55% of predicted) in SSc patients might characterize a peculiar SSc subset that may precede the development of PAH, and the progression of pulmonary vascular disease can be linked to decreasing DLCO trends [66,72,73,74]. 

## 8. Right Heart Catheterization 

**Figure 2:**
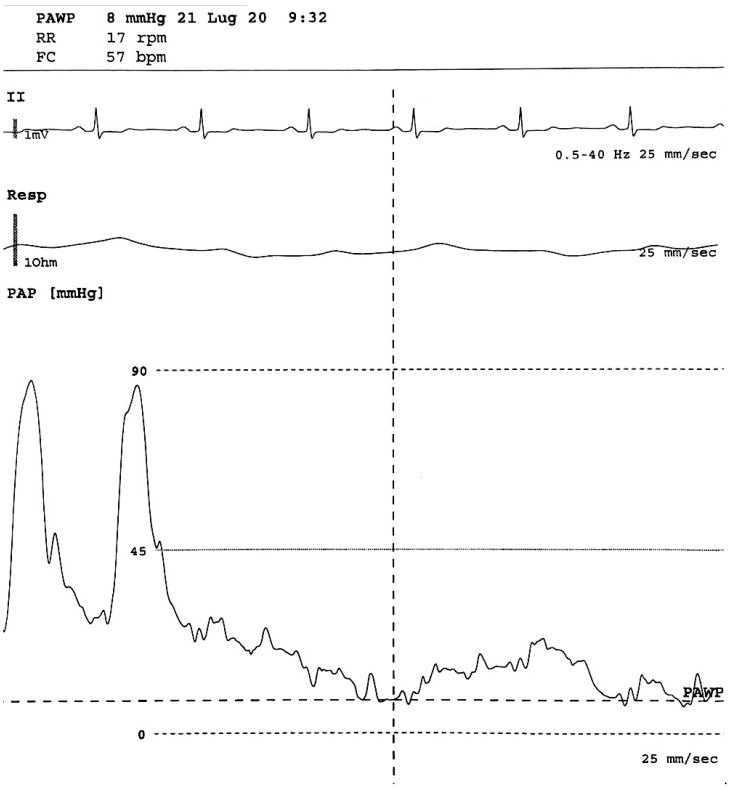
From below: pulmonary arterial pressure, respiratory and EKG waveforms during arterial catheterization. The first part of the pressure trace reflects the pressure in a pulmonary artery (large swings, dicrotic notch), then the balloon is inflated and the tip of the Swan Ganz catheter floats until it wedges in a small artery (small swings synchronous with respiratory rate), allowing a pulmonary arterial wedge pressure (PAWP) to be obtained, which is an indirect measure of left ventricle pressure.

Right heart catheterization (RHC) is the gold standard investigation for making a definitive diagnosis of pulmonary arterial hypertension (PAH) [80,81,82,83,84]. The RHC provides useful information on the degree of hemodynamic impairment, determines response to PAH therapy and establishes prognosis, providing information for clinical decision-making in PAH management [80,81,82,83,84] (Figure 2). Despite widespread acceptance, there are no internationally accepted clinical guidelines presenting the best practice for performing RHC. Therefore, to ensure the correct evaluation of directly measured hemodynamic or calculated parameters from RHC, procedures such as the position of the pressure transducer and catheter balloon inflation volume should be standardized with care [80,81,82,83,84]. The assessment of pulmonary arterial wedge pressure is particularly vulnerable to over- or under-wedging, which may lead to false readings. Moreover, errors in RHC measurement and data interpretation can complicate the differentiation of PAH from other disorders and lead to a misdiagnosis. Apart from diagnosis, the role of RHC in conjunction with non-invasive tests is on continuous expansion, encompassing the monitoring of treatment response and establishing the prognosis of patients diagnosed with PAH. However, it has been proposed that further standardization of RHC is warranted if we are to ensure its optimal use in routine clinical practice [80,81,82,83,84].

## 9. Peripheral Vascular Manifestations 

Raynaud’s phenomenon (RP), secondary to SSc, is the most frequent vascular manifestation in SSc patients. Secondary RP, the most common presenting feature of the disease, is observed in 95% of scleroderma patients and may precede diagnosis by many years [85,86,87,88]. During RP, the skin usually turns white (ischemia), blue (deoxygenation) and then red (reperfusion) [85,86,87,88].

Secondary Raynaud’s phenomenon (SRP) occurs in response to cold temperature or emotional stress, in the setting of underlying vascular disturbance, and is often associated with digital pain and ischemic ulcers [89,90,91,92]. However, it may occasionally lead to gangrene with tissue loss or the need for digital amputation [89,90].

As there is an obliterative vasculopathy of the peripheral arteries and microcirculation in SRP, it often leads to critical ischemia in scleroderma. There is often a luminal narrowing of >75% of digital arteries due to underlying intimal fibrosis and luminal occlusion caused by thrombi [85,86,87,88]. Endothelial cell injury and activation lead to vascular dysfunction and vasospasm that may quickly obstruct the already limited blood flow of the vasculopathic digital arteries [41].

Conversely, primary RP (PRP) is an isolated finding without underlying pathology (idiopathic). The suggested criteria for PRP include symmetric attacks, the absence of tissue necrosis, ulceration or gangrene, the absence of a secondary cause, negative tests for antinuclear antibodies and a normal erythrocyte sedimentation rate [85,86].

As a diagnosis of PRP is made at a time when no underlying disease has yet been identified, predicting whether or when it may turn into SRP is a difficult task [93,94,95,96]. As NVC detects morphological microcirculation abnormalities, it is able to distinguish SRP from both PRP and healthy subjects [97,98,99,100]. Therefore, primary RP patients should be carefully followed-up by NVC so as to allow for an early detection of the first signs of any transition to the secondary form of RP in the most reliable manner [101,102,103,104]. 

## 10. Nailfold Videocapillaroscopy

Morphological signs that represent the microvascular damage can be observed in nailfold videocapillaroscopy (NVC) images in SRP patients; these alterations include giant capillaries, microhemorrhages, capillary loss, the presence of avascular areas and angiogenesis [105,106,107]. These sequential capillaroscopic changes are typical of the microvascular involvement observed in more than 95% of SSc patients and are described as an “SSc pattern” [93,94,95,105]. The nailfold capillaries in PRP patients usually have a normal shape without any specific alterations. Whilst the presence of abnormal capillaroscopic findings, i.e., giant capillaries and microhemorrhages, are diagnostic of the early NVC pattern of scleroderma microangiography [105], the NVC technique is able to identify three morphological patterns specific to various SSc stages (early, active and late patterns) [105]. As reported hereafter, the early NVC pattern is characterized by a few enlarged/giant capillaries and capillary microhemorrhages, no evident capillary loss and a relatively well-preserved capillary distribution [105]. The most frequent alterations in the active NVC pattern are giant capillaries and capillary microhemorrhages, with a moderate capillary loss and a mild disorganization of the capillary architecture. There is severe capillary loss with evident avascular areas and disorganization of the normal capillary array in the late NVC pattern [105]. NVC provides a quantitative assessment of the microvascular damage, i.e., a quantification of certain characteristics and a semi-quantitative scoring. The characteristic capillaroscopic diagnostic parameters, i.e., irregularly enlarged capillaries, giant capillaries, microhemorrhages and progression parameters, which include fewer capillaries, capillary ramifications and capillary architectural disorganization, can be scored from 0 to 3 according to increasing severity and have been combined to create a semi-quantitative scale [105].

In healthy and primary RP subjects, NVC evaluation is characterized by morphological/structural homogeneity, evidencing 10–12 capillaries per linear millimeter, morphology of the capillary to “U” or “hairpin shape” and diameters of capillary branches of <20 μm [93,94,95]. Although it is quite common to observe normal nailfold capillaries in primary RP, capillaries with efferent branch enlargement or tortuosity may also be present [93,94,95].

The European League Against Rheumatism (EULAR) Study Group on Microcirculation in Rheumatic Diseases (EULAR SG MC/RD) has recently reported a simple consensus definition to name a single capillary as “(ab)normal”. The authors tried to standardize and clarify the differences between scleroderma and non-scleroderma patterns, avoiding confusion caused by the various different definitions used to describe non-scleroderma abnormal capillary morphology (e.g., “ramifications”, “neoangiogenesis” or “meandering”) [28]. 

In conclusion NVC, which combines a microscope (with system that ranges from 50 × up to 500 × magnification) and a digital video camera, represents a method for an early diagnosis and follow-up of nailfold microangiopathy—one of the earliest signs of morphological damage and change in SSc—and is a non-invasive, user-friendly, well-accepted, accessible and portable tool [28]. 

That is why abnormal nailfold capillaroscopic images, i.e., “scleroderma patterns”, were included in the 2013 European League Against Rheumatism and American College of Rheumatology’s classification criteria for SSc [103]. Several studies have also demonstrated that NVC is a promising tool for the prediction of clinical complication markers of severity and progression of SSc organ involvement [49,50,51,52,53,54].

## 11. The Correlation Between Peripheral Vascular and Pulmonary Involvement 

**Figure 3:**
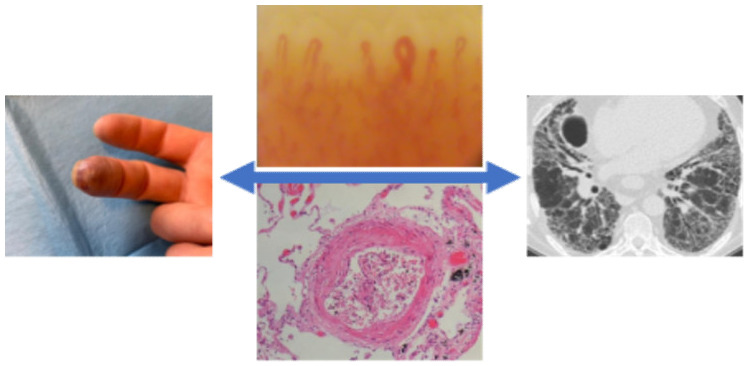
Vasculopathy in systemic sclerosis patients and the link between pulmonary damage and peripheral vascular manifestations.

Various studies have demonstrated that NVC alterations are associated with different SSc clinical complications and organ involvement [106,107,108,109,110,111,112]. Moreover, other authors reported on the correlation between NVC alterations and SSc-ILD diagnosed by HRCT [110,111]. Caetano et al., made a cross-sectional analysis of 48 SSc-ILD patients with HRCT and the presence of ground-glass opacities and/or fibrosis [111]. The same authors investigated the association between NVC findings, the presence and extent of ILD, as well as functional impairment. Capillary loss and avascular areas were significantly associated with the presence of ILD. The receiver operating characteristic (ROC) curve analysis confirmed the association between capillary loss and ILD (the area under the ROC curve, 90.1%; 95% CI, 81.8–91.4). Avascular areas and capillary loss were associated with a worse pulmonary function. No additional statistically significant difference was observed between ILD and other NVC findings (i.e., capillary dimension (*p*-value = 0.328), abnormal capillary morphology (*p*-value = 0.790) or the presence of hemorrhages (*p*-value = 0.187)) [111]. In another cross-sectional study, Guillen-del-Castillo et al. evaluated 134 SSc patients (58 with ILD on HRCT) with at least eight NVC (200 × magnification) images through both quantitative and qualitative examinations [110]. The SSc ILD patients had a lower median capillary density (4.86/mm vs 5.88/mm, *p*-value = 0.005) and higher median neoangiogenesis (0.56/mm vs 0.31/mm, *p*-value = 0.005). Moreover, more neoangiogenesis capillaries were observed in PAH patients (0.70/mm vs 0.33/mm, *p*-value = 0.008). A multivariate linear regression analysis emphasized a correlation between neoangiogenesis and decreased FVC (*p*-value < 0.001) and between the number of giant capillaries and reduced DLCO (*p*-value = 0.016) [110]. Guillen-del-Castillo et al., demonstrated that the late pattern was associated with lower FVC (*p*-value = 0.018) [110]. Jehangir et al.,’s case-control study made use of a dermascope to study the NVC pattern in 65 subjects: 10 patients with primary Raynaud’s phenomenon (RP), 40 with SSc and 15 age- and gender-matched controls. When testing HRCT and NVC patterns, only one patient with the early pattern had ILD, whereas those with an active or late pattern had a higher percentage of 55% and 100%, respectively [108] (Figure 3).

Markusse et al. performed NVC in 287 SSc patients aimed at assessing whether it could improve the detection of patients at high risk of cardiopulmonary involvement (82). The study population included 51% ILD patients, 59% with a DLCO decrease and 16% with a systolic pulmonary artery pressure (sPAP) of >35 mmHg. The NVC pattern showed a stable association with the presence of ILD or sPAP. The odds ratio (OR) for ILD was 1.3–1.4 (*p*-value < 0.05 for analyses with anti-RNAPIII, anti-RNP). The OR for DLCO was 1.5 (*p*-value < 0.05 for analyses with ACA, anti-Scl70, anti-RNAPIII, anti-RNP). The OR for sPAP was 2.2–2.4 (*p*-value < 0.05 for analyses with anti-RNAPIII, anti-RNP) [82].

It has been demonstrated that SSc-associated PAH is correlated with capillaroscopic changes identified by NVC [112,113,114,115,116,117,118,119,120,121,122,123,124,125,126,127]. NVC data are also markers of SSc severity and progression, such as reduced capillary density, which is associated with a high risk of developing PAH [112,113,114,115,116,117,118,119,120,121,122,123]. Some authors have supported the possibility of an early identification of a subset of patients with severe disease [112,113,114,115,116,117,118,119,120,121,122,123,124,125,126,127]. Hofstee et al. studied capillary density and dimensions and their association with pulmonary hemodynamic characteristics in 21 healthy controls, 20 idiopathic PAH patients and 40 SSc patients (21/40 had SSc-PAH, as determined by RHC) [123]. This study reported a significantly lower capillary density in SSc-PAH patients than in those without SSc-PAH (*p*-value = 0.001), although no statistically significant difference was observed for capillary dimensions (*p*-value > 0.05) [123].

Similarly, Corrado et al. evaluated 25 healthy subjects, 21 idiopathic PAH patients and 39 SSc patients (19/39 affected by SSc-PAH, determined by RHC, as mPAP ≥ 25 mmHg, PCWP ≤ 15 mmHg and pulmonary vascular resistance > 3 Wood units) [118]. The authors observed that the presence of PAH had a significantly inverse correlation with capillary density (*p*-value < 0.05) and correlated with both capillary dimension and giant capillaries (*p*-value < 0.05), which correlated with abnormal capillary morphology (*p*-value < 0.01) in SSc patients [118].

Riccieri et al.’s cross-sectional study evaluated NVC alterations in 12 consecutive SSc-PAH patients, confirmed by RHC. They demonstrated that NVC damage is correlated with the grade of PAH. 

Indeed, the NVC score (combining a semiquantitative score for density, dimension, presence of hemorrhage and morphology) and avascular area grading had a statistically significant correlation with PAH (*p*-value = 0.03 and *p*-value = 0.003, respectively) [121]. Furthermore, they observed that the active/late pattern was more common in SSc-PAH patients than in those without SSc-PAH (73% vs 50%, *p*-value < 0.05) [121]. This led to two recent meta-analyses confirming that microvascular changes detected by NVC are significantly associated with SSc-PAH, in particular lower capillary density and higher capillary width [112,117]. 

Kim et al. investigated the relationship between clinical manifestations and quantitative analysis of computerized NVC. They observed a strong correlation between capillary dimension and capillary loss with SSc-PAH (*p*-value < 0.05) and digital ulceration (*p*-value < 0.01) [114]. Moreover, a cross-sectional pilot study also assessed sublingual microvasculature by videocapillaroscope. The authors demonstrated that PAH patients had a lower sublingual microvasculature flow index and a higher vascular tortuosity than healthy age- and gender-matched control subjects [128].

In addition to the cross-sectional evidence, Smith et al.’s prospective longitudinal research studied 66 consecutive SSc patients. They defined the NVC pattern according to Cutolo’s classification and clinical evaluation was performed according to Medsger’s disease severity scale (DSS), with an 18–24 month follow-up [120]. They observed a statistically significant association between the NVC patterns and the development of future severe peripheral vascular or lung involvement. Indeed, the OR for future severe lung involvement, based on simple/multiple regression was 2.54/2.33 (*p*-value < 0.05) for early patterns, 6.43/5.44 for active patterns and 16.30/12.68 for late patterns. 

## 12. Conclusions

The characteristic features of SSc include extensive fibrosis, fibroproliferative vasculopathy and systemic autoimmunity (autoantibodies and T cell autoantigen reactivity). However, the vascular pathology in SSc is not necessarily an inflammatory process and may be better characterized as a vasculopathy. Some autopsy studies have shown that vasculopathy is a systemic process [128]. One such study is that of D’Angelo et al., who reported that SSc patients had widespread intimal proliferation in the pulmonary arteries [128]. Moreover, complex interactions between endothelial cells, vascular smooth muscle cells, extracellular matrix and circulating mediators contribute to vascular remodeling, vasospasm and vessel occlusion [128].

There is evidence to support the hypothesis that microangiopathy may be an important component of internal organ involvement and that NVC is a candidate biomarker for the assessment of pulmonary damage. 

There are numerous reports finding a good correlation between distinctive quantitative and qualitative NVC features and the presence of ILD on HRCT, as well as lung functional parameters such as FVC and DLCO. Therefore, it might well be that microangiopathy is a pivotal process in the establishment and progression of SSc-ILD. Similarly, as NVC is capable of detecting the early microvascular changes associated with the presence of PAH, it may well play a significant role in early prediction of SSc-PAH.

The gold standard with which to diagnose SSc-ILD is still the chest HRCT, whilst RHC is the validated test with which to diagnose PAH. Due to the poor prognosis for SSc patients with organ damage, in particular those with pulmonary manifestations, all these patients should be carefully evaluated from the early disease phase and followed-up. This should hopefully facilitate early identification and the choice of an early appropriate therapeutic regimen. 

This review reports on the different clinical manifestations and tests, i.e., clinical, radiological and pulmonary function tests that may be used for the early prediction of lung involvement in SSc patients. It also includes evidence from the literature on how NVC may also be a promising tool for early identification and/or prediction of pulmonary complications. The data herein reported support the possibility that NVC might be incorporated together with other parameters in high-performance algorithms (e.g., DETECT algorithm for SSc-PAH, ILD screening procedures) in the early detection of lung involvement in SSc. 

## Data Availability

Not applicable.
